# Unraveling the Genomic Evolution of Dengue Virus Serotype 1: A Case Study from Yantai, China

**DOI:** 10.3390/life14070808

**Published:** 2024-06-26

**Authors:** Yulou Sun, Liqun He, Xia Li, Cong Li, Shicui Yan, Yi Zhang, Zhenlu Sun

**Affiliations:** Yantai Center for Disease Control and Prevention, Yantai 264003, China; sunyulou90@gmail.com (Y.S.); hlq0831@163.com (L.H.);

**Keywords:** dengue fever, dengue virus, whole genome sequence, Bayesian evolutionary analysis, serotype, genotype

## Abstract

In August 2023, we identified a case of dengue fever in Yantai City, which was imported from Xishuangbanna, China. To investigate its evolutionary history and population dynamics, we utilized the metatranscriptomic method to obtain the virus’ whole genome sequence. Together with 367 selected dengue virus whole genome sequences from the NCBI database, we constructed a time-scaled Maximum Clade Credibility (MCC) tree. We found that our sequence exhibited a high homology with a sequence of DENV1 (OR418422.1) uploaded by the Guangzhou Center for Disease Control and Prevention in 2023, with an estimated divergence time around 2019 (95% HPD: 2017–2023), coinciding with the emergence of SARS-CoV-2. The DENV strain obtained in this study belongs to genotype I of DENV1. Its ancestors experienced a global epidemic around 2005 (95% HPD: 2002–2010), and its progeny strains have spread extensively in Southeast Asia and China since around 2007 (95% HPD: 2006–2011). The Bayesian skyline plot indicates that the current population of DENV1 has not been affected by SARS-CoV-2 and is expected to maintain stable transmission. Hence, it is imperative to track and monitor its epidemiological trends and genomic variations to prevent potential large-scale outbreaks in the post-SARS-CoV-2 era.

## 1. Introduction

Dengue fever (DF) is a severe infectious disease caused by the dengue virus (DENV), which poses a significant public health challenge globally [1,2]. According to the World Health Organization (WHO), dengue is the fastest-spreading mosquito-borne viral disease globally. From January to April 2024, over 90 countries have reported dengue fever outbreaks, a significant rise from the 1970s when only nine countries were affected. The spread has been accelerated by increased urbanization, climate change, and international travel [3,4]. In this four-month span, there were more than 7.6 million reported cases, including over 16,000 severe cases and more than 3000 deaths (https://worldhealthorg.shinyapps.io/dengue_global/, accessed on 30 April 2024). Given the current outbreak scale, the potential spread risk, and the complex transmission factors, the overall risk at the global level is assessed as being High by the WHO. Notably, the epidemic’s impact is most profound in impoverished and underdeveloped regions, which lack robust detection and reporting mechanisms [2]. As a result, the global burden of dengue fever is severely underestimated. DENV, a single-stranded positive-sense RNA virus, is classified within the Flaviviridae family, Flavivirus genus, and Dengue virus species [3,5]. Its genome is approximately 11,000 bp long and contains an open reading frame (ORF) encoding three structural proteins and seven nonstructural proteins [6,7,8,9]. To date, four distinct serotypes have been identified—DENV-1, DENV-2, DENV-3, and DENV-4 [10,11,12,13,14,15]. Each serotype contains various genotypes, with no more than 6% differences in the nucleotide sequence [3,16,17]. Specifically, DENV-1 has five genotypes (I, II, III, IV, and V) [18].

The symptoms of human infection with DENV exhibit a broad spectrum, ranging from asymptomatic infections to mild or severe clinical manifestations. Due to the large number of undetected infections, the exact incidence rate of DF is difficult to determine, and the actual number of infections is significantly higher than reported statistics [1]. Initial infection with a certain serotype of DENV typically results in an asymptomatic infection or manifests as mild DF, characterized by biphasic fever, myalgia, headache, arthralgia, retro-orbital pain, rash, thrombocytopenia, lymphadenopathy, and leukopenia [19]. The generated antibodies not only lack sufficient cross-protection against other serotypes, but also lead to the antibody-dependent enhancement (ADE) of the viral infection [20,21,22,23,24]. A subsequent infection with a different serotype of DENV can result in severe DF, with symptoms including dengue hemorrhagic fever (DHF), characterized by hemostatic dysfunction and an increased vascular permeability, as well as a hypovolemic shock known as dengue shock syndrome (DSS) [25].

DF is a mosquito-borne disease that predominantly affects tropical and subtropical countries and regions, with the primary and secondary vectors being Aedes aegypti and Aedes albopictus, respectively. [2,26,27]. Although China is not a major endemic country, a substantial number of cases are reported annually. In 2023, China reported a total of 19,627 DF cases, including one fatal case (https://www.ndcpa.gov.cn/). DENV-1 is the most prevalent serotype, with Genotype I being the dominant lineage [16]. In China, both vectors are present. Aedes aegypti has a stronger transmission capability but a limited distribution range. Conversely, Aedes albopictus has a widespread distribution and is the main vector for dengue transmission in China. Yantai City was not a traditional endemic area for DF. However, due to frequent population movements, sporadic cases of DF have been identified in recent years [16]. Concurrently, Aedes albopictus is extensively distributed in Yantai City, posing a risk for the local transmission of DENV [28]. In August 2023, an inter-provincial imported case of DF was detected in Yantai City. Subsequently, we obtained the full viral genome sequence and conducted a study on its population evolution.

## 2. Materials and Methods

### 2.1. Sample Collection

The sample for this study was derived from the serum of a suspected case of DENV infection identified in Yantai City, China, in August 2023. The case is a 34-year-old female without acute or chronic disease history. The case presents with symptoms such as fever, headache, nausea, and vomiting. The blood routine examination showed that the white blood cell count (WBC) was 2.88 × 10^9^/L, the red blood cell count (RBC) was 4.35 × 10^12^/L, the absolute neutrophil count (ANC) was 1.99 × 10^9^/L, hemoglobin (Hb) was found at 143.00 g/L, hematocrit (HCT) was 43.1%, and hypersensitive C-reactive protein (hs-CRP) was found at 1.92 mg/L. Through its travel history, we discovered that the case had lived in Xishuangbanna and suffered mosquito bites. During this time, the case’s friend received a diagnosis of DENV infection, and we suspected that the case may also be infected with DENV. Therefore, we took a serum sample from this case and conducted nucleic acid tests. This method aims to confirm DENV infection by directly detecting the virus’s nucleic acid.

### 2.2. Virus Detection and Sequencing

According to the manual instructions, the QIAamp viral RNA micro kit (Qiagen, Hilden, Germany, Cat No. 52904) was used to extract viral RNA from 140 μL of blood serum. The purified RNA was subsequently eluted in 50 µL of elution buffer. Fluorescent qPCR was performed using an In Vitro Diagnostic (IVD) reagent kit (Beijing Applied Biological Technologies Co., Ltd., Beijing, China, Cat No. A3801-50T) in a 25 μL reaction following the manufacturer’s instructions. The kits’ detection threshold was established at 1.0 × 10^3^ copies/mL, with a cycle threshold (CT) value below 36 indicating a positive result. For this case, the CT value we obtained was 26.5, denoting a robust positive signal.

In order to obtain the whole genome sequence of DENV, a metatranscriptomic approach was employed using the VAHTS Universal V8 RNA-seq Library Prep Kit (Cat No. NR605-C2) for Illumina, a product of Vazyme (Nanjing Vazyme Biotech Co., Ltd., Nanjing, China). The library’s quantification and validation were performed using the Qubit 4.0 Fluorometer system (Life Technologies, Carlsbad, CA, USA, Cat No. Q33231) and the 2100 Bioanalyzer (Agilent Technologies, Santa Clara, CA, USA, Cat No. 5067-4626), respectively. Subsequent sequencing of the library was performed on the NextSeq2000 platform using NextSeq2000 P1 Reagents (300 cycles; Illumina, San Diego, CA, USA, Cat No. 20049920).

### 2.3. Sequence Data Analysis

We first assessed the quality of the raw data using FastQC software (Babraham Institute, Cambridge, UK), and imported these raw data into CLC Genomics Workbench 22.0.2 (Qiagen, Hilden, Germany). Then, we conducted taxonomic profiling using the Clustered Reference Viral Database (RVDB) (21 June 2021), which was constructed by CLC teamwork. Next, we extracted reads belonging to dengue by setting the ‘Aggregate feature’ parameter to ‘Species’. In order to identify an accurate reference sequence for subsequent alignments, we extracted all dengue-related sequences from the RVDB database to form a reference data set. Then, we aligned the previously obtained reads to this reference data set using the ‘Find Best Matches using K-mer Spectra’ tool within the CLC Genomics Workbench. Then, we obtained one optimal sequence (GeneBank ID: FJ906965.1) that belongs to serotype 1 as the reference sequence. We performed subsequent analyses using the ‘Resequencing Analysis’ modules within the CLC Genomics Workbench to obtain the whole genome sequence. The steps included the following: (1) Aligning the raw data with the reference sequence FJ906965.1. (2) Deriving a consistent sequence from the regions with a depth ≥ 3, while regions with a depth < 3 were replaced with ‘N’.

### 2.4. Bayesian Evolutionary Analyses

In order to analyze the origin of these sequences and explore the global population expansion of dengue type 1, we employed the BEAST2 (v2.7.5) software suite for Bayesian phylogenetic analysis [29]. The Maximum Clade Credibility (MCC)-dated phylogenetic tree was constructed using the Bayesian Markov chain Monte Carlo (MCMC) approach. Compared with the E gene, the whole genome sequence covers more comprehensive mutation information and can obtain more accurate results [30]. We downloaded all the whole genome sequences of dengue genotype 1 in the NCBI database (https://www.ncbi.nlm.nih.gov/) and we performed an initial screening to discard any sequence that was incomplete or lacked essential information. To reduce redundancy and over-representation, we used CD-Hit-EST v4.8.1 software to cluster sequences with 100% similarity [31]. Subsequently, to simplify the data set, we re-clustered the sequences based on their country of origin and the date of isolation. From each cluster, only one accession was randomly selected. Following this, we made two critical adjustments to the data set.

Firstly, we identified and excluded such recombination sequences using several methods within the RDP4 v4.101 software, including RDP, GENECONV, MaxChi, BootScan, and SiScana [32]. Secondly, to evaluate the temporal structure within the sequence data, we regressed phylogenetic root-to-tip distances against the sampling dates using TempEst v1.5.3 [33]. As its input, TempEst requires a ‘nonclock’ phylogenetic tree, so we used IQ-TREE to build an ML tree with a bootstrap value set to 1000 [34]. During the temporal structure analysis, we examined the data for outliers and removed them. In this step, we identified sequences, and replaced these and previously detected recombinant sequences with alternative random sequences from the same year and country. Finally, we reconfirmed the absence of anomalies and recombinants within the data set to ensure that the final data set did not contain any anomalous or recombinant sequences.

To determine the most suitable molecular clock model and tree prior for our analysis, we employed path sampling to compare marginal likelihoods. We selected two molecular clocks (Strict clock and Optimized Relaxed Clock) and three tree prior models (Coalescent Bayesian skyline, Coalescent constant population, and Coalescent exponential population) for combined analysis. We specified the number of path steps as 10, with each chain having a length of 10 million iterations. The outcomes of the comparison are shown in Table 1.

Coalescent Bayesian skyline tree priors and Optimized Relaxed Clock provided the best fit to our data sets, and this combination was chosen for subsequent analysis. In order to achieve reasonable Effective Sample Sizes (ESSs > 200), we set the chain length to 300 million and logged every 30,000. The BEAGLE v4.0.0 parallel computation library was utilized to speed up the analysis process [35]. To determine if there was enough temporal signal in the data set for calibration, we used the R package TipDatingBeast for a date-randomization test [36]. Once the analysis was finalized, we used Tracer v1.7.2 to check for sufficient sampling and convergence after discarding 10% as burn-in [37]. We generated the Maximum Clade Credibility (MCC) tree using the TreeAnnotator program within BEAST, and visualized it using Figtree v1.4.4 (http://tree.bio.ed.ac.uk/software/figtree, accessed on 30 April 2024). Furthermore, we constructed the Bayesian Skyline plot with the Tracer software.

## 3. Results

**Figure 1:**
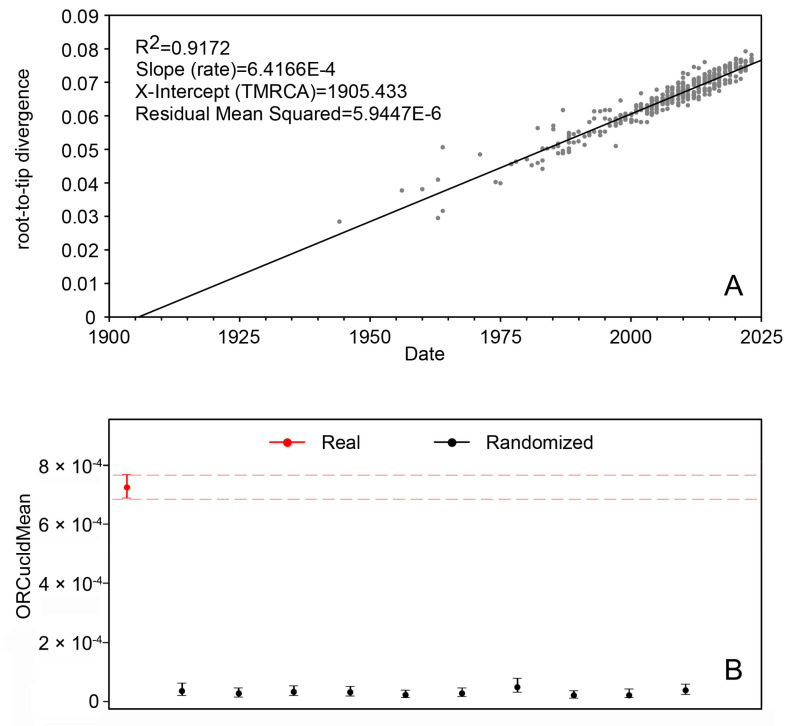
Plot for root-to-tip divergence and date-randomization test. (**A**) A linear regression plot for root-to-tip divergence versus sampling year. (**B**) Date-randomization test performed on ORCucldMean.

A total of 368 DENV1 whole genome sequences were included in this study, including the following four genotypes: I, II, IV, and V. Notably, genotype III is a sylvatic type and was not included in this study [30]. When performing the recombination analysis, we found that the sequence we obtained did not undergo recombination. The subsequent temporal structure analysis yielded an R^2^ value of 0.9172, which indicates that the data have a strong temporal structure (Figure 1A). Additionally, date-randomization testing revealed sufficient time signals in the data set for time calibration (Figure 1B).

**Figure 2:**
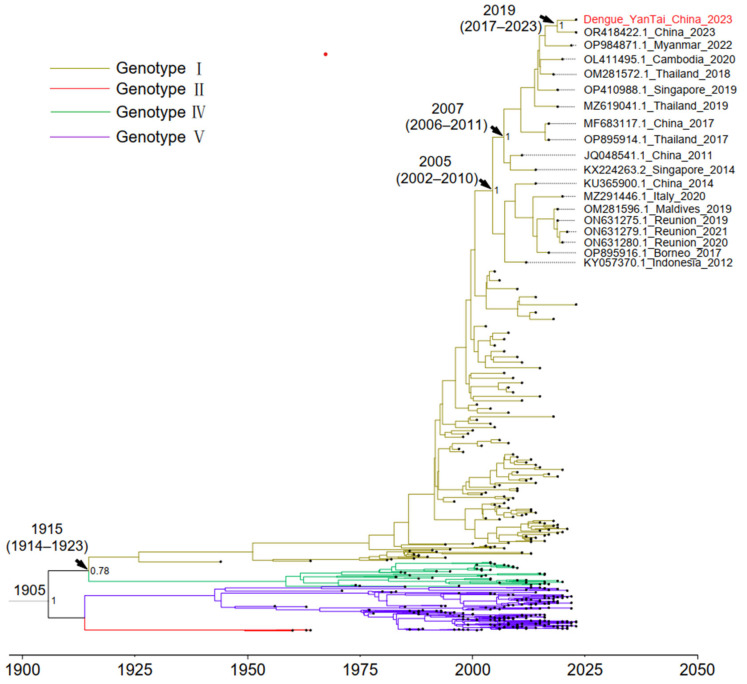
Time-scaled Maximum Clade Credibility trees inferred from the DENV1 whole genome sequences. The scale axis is representative of calendar years. Arrows represent key node divergence times (95% HPD), and the posterior probability was expressed behind the node. Red fonts represent the strain sequenced in this study.

**Figure 3:**
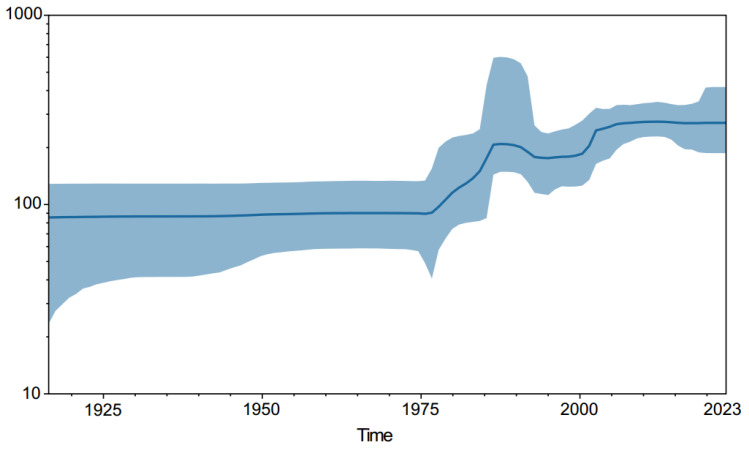
Bayesian skyline plots of the DENV1 whole genome sequences. The *x*-axis shows the time, and the *y*-axis shows the log of the effective population size. The solid line represents the median estimates of population size, and the shaded area indicates the 95% HPD intervals.

According to the results of Bayesian evolutionary analysis (Figure 1), the time to Most Recent Common Ancestor (TMRCA) of the four genotypes of DENV1 is around 1905. The divergence between genotypes I and IV occurred in 1915 (95% Highest Posterior Density [HPD]: 1914–1923) (Figure 2), which is effectively consistent with the results of a previous study by Stata et al. (1909, HPD: 1896–1919) [30]. Genotypes I, IV, and V were all endemic in China, and the strain in this study belongs to genotype I. Although the patient came from Xishuangbanna, the whole genome sequence of the virus was highly homologous to the sequence of a DENV1-I strain (OR418422.1) uploaded by the Guangzhou Center for Disease Control and Prevention in 2023. The divergence time between the two sequences is probably in 2019 (95% HPD, 2017–2023). Our retrospective analysis of the timeline revealed that this lineage underwent a significant evolutionary divergence around 2005 (95% HPD: 2002–2010). Following this divergence, its descendants proliferated across the Asian region around 2007 (95% HPD: 2006–2011), predominantly in Southeast Asia and China. In order to assess the population size changes of DENV1, we constructed a Bayesian skyline plot (Figure 3). The plot reveals a substantial expansion of the DENV1 population between 1975 and 2000. However, entering the 21st century, the population size seems to have stabilized.

## 4. Discussion

In this study, we used high-throughput sequencing technology to obtain the complete genomic sequence of a DENV from a case imported from Xishuangbanna to Yantai City, China. By comparing the obtained sequence with global DENV1 sequences, we unveiled the evolutionary history and population dynamics of the ancestral sequences. Phylogenetic analysis indicated that this strain in our study belongs to genotype I of serotype I. Its ancestor underwent a global epidemic around 2005 (95% HPD: 2002–2010), and its descendant strains have been widely circulating in China and Southeast Asia since 2007 (95% HPD: 2006–2011). This aligns with previous studies, which show an extensive spread of DENV1 in Southeast Asia and China since the early 21st century [38].

The sequence we identified showed a high homology with a sequence reported by the Guangzhou Center for Disease Control and Prevention in 2023, with an estimated divergence time around 2019 (95% HPD: 2017–2023). Notably, in 2019, China experienced a major dengue outbreak, affecting 13 provinces and resulting in a total of 22,599 confirmed cases, significantly higher than the 5136 cases reported in 2018 [39]. Both Xishuangbanna and Guangzhou also reported peak dengue outbreaks in 2019, with Xishuangbanna reaching a historical high of 3,931 cases and Guangzhou reporting its second-highest number of cases at 1634 [40,41]. Combined with the results of this study, it indicates that the descendant strains of the 2019 dengue outbreak continue to circulate widely in southern China.

Bayesian skyline plots revealed a substantial expansion in the population size of DENV1 around 1975, correlating with global population growth, industrialization-induced climate warming, and increased global interactions during that period [30]. Interestingly, the population size of DENV1 remained stable throughout the SARS-CoV-2 pandemic (2019–2023). Despite strict containment measures enforced globally during this period, it appears that the transmission of DENV remained unaffected. This is primarily due to the different transmission routes of DENV and SARS-CoV-2. SARS-CoV-2 primarily spreads via respiratory droplets and direct contact, which can be effectively controlled through measures such as mask wearing and social distancing. In contrast, DENV is primarily transmitted by mosquitoes (notably Aedes aegypti and Aedes albopictus), and factors such as urban density, climate conditions, and vector habitat suitability continue to support the transmission of dengue fever even during the global health crisis [42,43,44,45]. Therefore, it is crucial to monitor epidemiological trends and strengthen vector management to prevent large-scale dengue virus outbreaks in the post-COVID-19 era [46].

## 5. Conclusions

Yantai, a port city located in a temperate zone, is not traditionally considered as an endemic region for DF. However, it is a prevalent region for the virus’s vector, Aedes albopictus [28]. With global warming, accelerated urbanization, and increased internationalization, the geographical range of virus transmission is expected to expand [47]. As early as 2017, Jining City, in the same province, experienced a local dengue outbreak, which means the risk of dengue outbreaks in Yantai remains [48]. The potential domestic transmission routes revealed by our study indicate the necessity for coordinated efforts across different regions of China. Effective vector control measures, public health education, and robust diagnostic capabilities are crucial to mitigating the risk of large-scale outbreaks. This study provides a complete set of methods for analyzing the genomic evolution of DENV, which can not only serve as a guide for future research, but can also expand to the study of other viruses. This is of great significance for epidemic prevention, control, and public health.

## Figures and Tables

**Table 1:** Marginal likelihoods of different combinations of clock model and tree prior.

Molecular Clock Model	Coalescent Tree Prior	Log Marginal Likelihood
Strict clock	Coalescent Bayesian skyline	−138,201.16
Strict clock	Coalescent constant population	−138,201.07
Strict clock	Coalescent exponential population	−138,198.30
Optimized Relaxed Clock	Coalescent Bayesian skyline	−137,786.76
Optimized Relaxed Clock	Coalescent constant population	−137,795.23
Optimized Relaxed Clock	Coalescent exponential population	−137,798.36

The best-fitting tree prior and molecular clock model are indicated in red font.

## Data Availability

The original contributions presented in the study are included in the article, further inquiries can be directed to the corresponding authors.
